# Droplets Patterning of Structurally Integrated 3D Conductive Networks-Based Flexible Strain Sensors for Healthcare Monitoring

**DOI:** 10.3390/nano13010181

**Published:** 2022-12-30

**Authors:** Yang Zhang, Danjiao Zhao, Lei Cao, Lanlan Fan, Aiping Lin, Shufen Wang, Feng Gu, Aibing Yu

**Affiliations:** 1 Laboratory of Advanced Materials and Manufacturing (LAMM), Nanchang Key Laboratory for Advanced Manufacturing of Electronic Information Materials and Devices, International Institute for Innovation, Jiangxi University of Science and Technology, Nanchang 330013, China; 2 Institute for Process Modelling and Optimization, Jiangsu Industrial Technology Research Institute, Suzhou 215123, China; 3 Department of Chemical Engineering, Monash University, Melbourne, VIC 3800, Australia

**Keywords:** strain sensors, flexible devices, hybrid nanostructures, aerosol jet printing, wearable electronics

## Abstract

Flexible strain sensors with significant extensibility, stability, and durability are essential for public healthcare due to their ability to monitor vital health signals noninvasively. However, thus far, the conductive networks have been plagued by the inconsistent interface states of the conductive components, which hampered the ultimate sensitivity performance. Here, we demonstrate structurally integrated 3D conductive networks-based flexible strain sensors of hybrid Ag nanorods/nanoparticles(AgNRs/NPs) by combining a droplet-based aerosol jet printing(AJP) process and a feasible transfer process. Structurally integrated 3D conductive networks have been intentionally developed by tweaking droplets deposition behaviors at multi-scale for efficient hybridization and ordered assembly of AgNRs/NPs. The hybrid AgNRs/NPs enhance interfacial conduction and mechanical properties during stretching. In a strain range of 25%, the developed sensor demonstrates an ideal gauge factor of 23.18. When real-time monitoring of finger bending, arm bending, squatting, and vocalization, the fabricated sensors revealed effective responses to human movements. Our findings demonstrate the efficient droplet-based AJP process is particularly capable of developing advanced flexible devices for optoelectronics and wearable electronics applications.

## 1. Introduction

Flexible sensors, which can be used for the detection of human movement, electronic skin, human physiological signals, temperature monitoring, and other things, have sparked a great deal of interest with the development of flexible electronics [1,2,3,4,5]. As a type of flexible sensor, flexible strain sensors are able to transform external mechanical deformation into a point signal used to monitor human movement to expedite health evaluations and disease diagnosis [6,7]. Common types of flexible strain sensors are resistive and capacitive, with resistive sensors being the first to be commercially accessible and the change in resistance being very simply monitored by electrical measurement [8]. When developing flexible strain sensors with considerable extensibility, stability and durability, the sensitive performance should be optimized at multi-scales [9]. The interface state of the components associated with the positional shift generated by stretching or releasing determines the change in resistance, whereas the constructed networks determine the conductive pathways [10].

A variety of electrode materials, including silver nanowires (AgNWs), silver nanoparticles (AgNPs), silver nanorods (AgNRs), graphene, MXene, carbon nanotubes, PEDOT:PSS, and others, are frequently utilized to create flexible strain sensors [11,12]. As compared to conductive polymers and carbon-based materials, metal-based compounds are more reliable and offer better conductivity [13]. Due to their high electrical conductivity and flexibility, one-dimensional AgNWs have been tentatively exploited for flexible strain sensors besides their applications in constructing flexible light-emitting diodes, solar cells, and transparent conductive films, etc [14,15,16]. For AgNWs-based strain sensors, the sensing behaviors are strongly associated with the mutual overlap of nanowires, which provides conductive pathways [17]. However, the as-reported AgNWs-based flexible strain sensors exhibit disordered gaps between their layers and the interfaces were in random states, resulting in inconsistencies in strain sensor performance [18,19]. By introducing AgNPs, the mutual overlap of the AgNWs/AgNPs composites could be somewhat optimized with enhanced sensitivity; nevertheless, the interfaces between these conductive components could not be manipulated in situ using conventional techniques [20]. In light of this challenge, these inconsistencies should be purposefully assembled Ag components at multi-scales to create structurally integrated conductive networks with hybrid interfaces, but this has proven to be exceptionally difficult to achieve.

Droplet-based printing electronic technology is an emerging technique that drastically lowers the cost of conventional silicon technologies and allows for the quick fabrication of flexible electronics [21,22]. Aerosol jet printing (AJP) is competitive among various droplet-based methods because of its wide selection of inks and materials, minimum feature size of 10 μm, good substrate compatibility, and coating uniformity [23,24,25]. By delivering a multiple quasi-parallel aerosol droplets flow, comparable to a conical fibers array (CFA)-guided liquid transfer process, the AJP technique has been recognized as one of the best methods for patterning micro-scale droplets for ordered networks generation [26]. Different driving forces would be generated to determine the deposition behaviors of the suspended non-volatile solutes in the pinned or spreading droplets by engineering wetting, dewetting, as well as the coalescence of the droplets [27]. With complete solution evaporation, the relative strength of capillary flow and Marangoni reflux would compete to determine the parallel pattern topology, with sap flow transporting the solutes outward [28,29]. In particular, the aerosol droplets are anticipated to function as the best available microreactor for fostering the reactions that would form nanostructures inside the droplets [30]. By regulating the coalescence and microreaction of parallel droplets, MXene nanosheets have recently been curled and aligned [31].

Herein, an innovative droplet-based aerosol jet printing method has been devised by combining a feasible transfer process with the aim of fabricating structurally integrated 3D conductive networks of hybrid AgNRs/NPs for flexible strain sensors. The structurally integrated 3D conductive networks could be intentionally developed by tweaking the droplet deposition behaviors as well as their assembly with multiple orthogonal printing passes. When stretched, the conductive 3D networks encased in polyvinyl alcohol (PVA) elastomer promote slippage between materials, resulting in an obvious change in resistance of the sensors. Under varied tensile circumstances, the sensing behaviors of strain sensors comprising different layers were investigated. In a strain range of 25%, the 15-layered sensor has the optimal gauge factor of 23.18. By applying the sensors to real-time monitoring of finger bending, arm bending, squatting and vocalization, it was capable of demonstrating an effective response to human movement. This study highlights the great potential of AJP for the fabrication of flexible devices for next-generation applications in optoelectronics and wearable electronics, with the capability of structurally integration of materials at the microscale intentionally.

## 2. Materials and Methods

### 2.1. Materials

Polyvinyl alcohol (PVA), Polyvinylpyrrolidone (PVP), FeCl_3_ and NH_4_Br were purchased from Aladdin Reagent (Shanghai) Co., Ltd., Shanghai, China, and used as received. Ethylene glycol (EG) was purchased from Shanghai Macklin Biochemical Co., Ltd., Shanghai, China, and used as received. AgNO_3_ was supplied by Sinopharm Chemical Reagent Co., Ltd., Shanghai, China, and used as received. The silver paste was provided by Suzhou’s Test Electronic Technology Co., Ltd, Suzhou, China.

### 2.2. AgNWs Synthesis and Ink Formulation

The precursor AgNWs were synthesized via a modified polyol technique [32]. PVP (MW = 58,000, 0.64 g) and AgNO_3_ (0.72 g) were first dissolved in 85 and 15 mL of EG, respectively. After completely dissolving, the two solutions were mixed. 8 mL of FeCl_3_ EG solution (600 μM) and 6 mL of NH_4_Br EG solution (600 μM) were added, followed by 5 min of stirring in an oil bath at 130 °C. Then, the solution was placed in an oil bath at 130 °C for 4 h without being stirred. After being centrifugal cleaning with deionized water and ethanol, the obtained precipitate was dispersed in 20 mL of deionized water as the precursor inks for use.

### 2.3. Fabrication of the Strain Sensor

A commercial aerosol jet printer (HMP, WE Electronics (Suzhou) Technology Co., Ltd., Suzhou, China) was utilized to print the 3D networks of hybrid Ag species. The patterns were designed using the AutoCAD software (Autodesk Inc., San Francisco, CA, USA) in a printer-readable.dxf format with two grid squares (5 mm × 5 mm) linked by a single long strip of grids (20 mm × 3 mm). The deposition temperature was set at 75 °C. The ink of precursor AgNWs was first aerosolized by applying an ultrasonic atomizer (1.7 MHz). The generated aerosol stream was suspended by N_2_ as a carrier gas to the nozzle and further constrained by a sheath gas of N_2_ before being jetted onto the clean glass substrate. Alternative orthogonal printing passes was used to create 3D networks. The nozzle was 300 μm in diameter, while the stand-off height was 5 mm. During printing, the printing speed was 10 mm s^−1^. 

Following that, a PVA solution was cast onto the printed patterns with a tetrahedral coating preparation device. The PVA solution was prepared by dissolving PVA (5 g) in deionized water (50 mL). After stirring at 85 °C for 3 h, the obtained transparent solution was used for the experiments. The film was peeled away from the glass substrate after 12 hours. Ultimately, the strain sensors were manufactured by attaching the conductive adhesive tapes and squares on both sides of the printed patterns with commercial silver paste.

### 2.4. Characterization

The structural morphology of the sample was characterized by a SEM (Zeiss Sigma 300, Carl Zeiss Microscopy Ltd., Cambridge, UK) and a TEM (Titan G260-300, Frequency Electronics, Inc., Hillsboro, OR, USA). The UV absorption spectrum of the ink was measured by an UV-Vis spectrophotometer (UV-2600, Shimadzu Corporation, Tokyo, Japan). The system of an electrochemical workstation (CH CHI660E, CH Instruments, Inc., Austin, TX, USA) was used for the real-timely recording of the change of electrical properties. The resistance of the sensors was measured via a multimeter (VC86E, Bei Cheng (Hong Kong) Technology Co., Ltd., Shenzhen, China). Prtronic flexible electronic tester was used for the cyclic tensile tests (FT2000, Shanghai Mifang Electronic Technology Co., Ltd., Shanghai, China). The optical image of the printed patterns was achieved with an optical microscope (DM6 M, Leica Microsystems, Mannheim, Germany).

## 3. Results and Discussion

### 3.1. Structurally Integrated 3D Conductive Network

For droplet-based printing technologies, the formulation of the ink is typically crucial for defining the print quality [32]. AJP is advantageous in these droplet-based technologies because of its low ink formulation requirements, which can be satisfied by dispersing or dissolving materials in solutions without additives [33]. In this study, the ink was formulated by dispersing AgNWs directly in deionized water (Figure S1a). The AgNWs were synthesized successfully using a modified polyol technique [34]. The presence of two characteristic absorption peaks in the UV absorption spectrum suggests that the formation of AgNWs is homogenous (Figure S1b) [35]. According to previously published works, the introduction of mediated agents had a significant impact on the formation of AgNWs. For instance, Cl^−^ ions promote the formation of AgNWs, while Br^−^ ions significantly reduce the diameter of AgNWs and Fe^3+^ ions restore the dissolved oxygen to balance the etching [36]. In this work, the length and diameter of the formed AgNWs are less than 40 μm and 100 nm (Figure S1c,d), respectively. During the AJP procedure, the ink was atomized via ultrasonication, which has been demonstrated to fragment AgNWs into nanorods and nanoparticles (Figure S1e) [37]. The ultrasonication may create cavitation bubbles with huge concentrations of energy, which are delivered to the AgNWs as powerful bubble jets with flow rates up to 1000 m s^−1^ and pressures up to 1 GPa [38]. As a result, the AgNWs are fragmented into small fragments, such as nanorods and nanoparticles, which will serve as the building components for the 3D networks. From the scanning electron microscope (SEM) image, the fragmented particle size is estimated to be 100 nm and the length of the fragmented Ag nanorod is less than 5 μm while the diameter is consistent with its precursor nanowires (Figure S1e,f).

**Figure 1:**
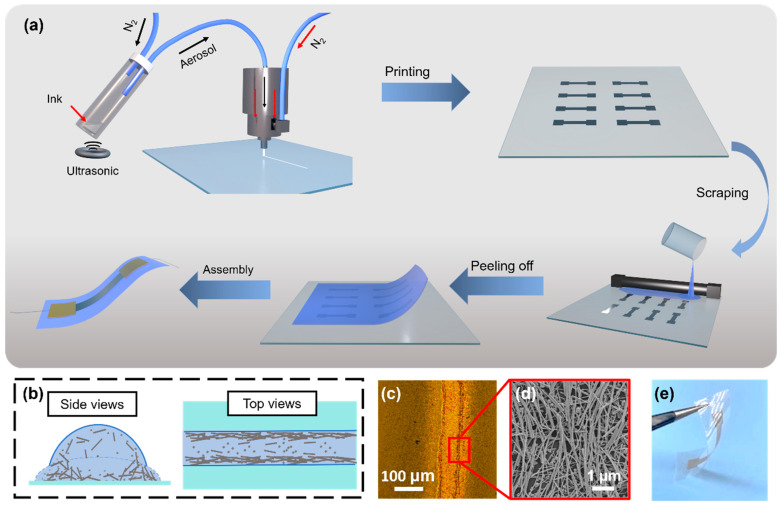
Fabrication of structurally integrated 3D conductive networks of hybrid AgNRs/NPs. (**a**) Schematic of fabrication procedure for 3D conductive networks of hybrid AgNRs/NPs embedded in a polymer matrix. (**b**) The dewetting model of deposited quasi-parallel droplets showing Ag species migrating outward and assembling orientedly. (**c**) Optical image of the printing pattern showing coffee-ring effect. (**d**) SEM image of oriented assembly of Ag species at the pattern edge. (**e**) Photograph of the elastomer-encased 3D networks.

**Figure 2:**
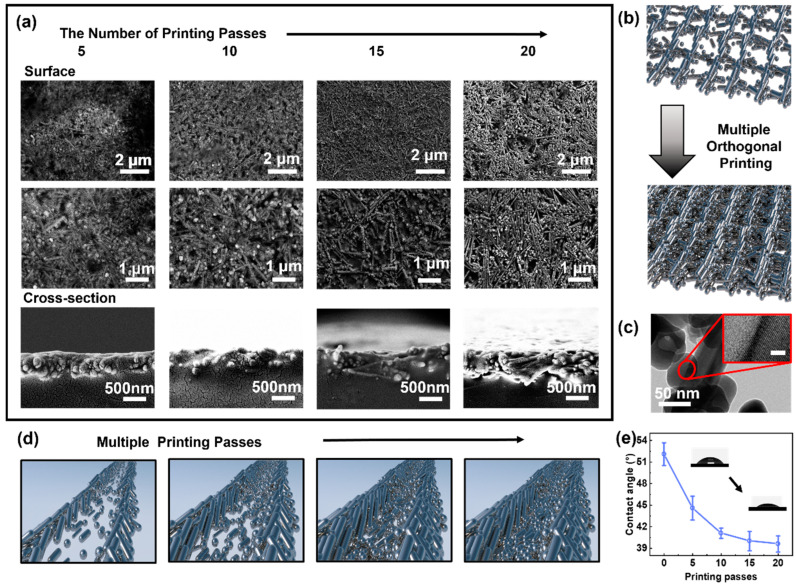
Morphology and microstructure of the structurally integrated 3D conductive networks of hybrid AgNRs/NPs. (**a**) SEM and cross-sectional SEM images of the elastomer-encased 3D networks with different print layers. (**b**)The fabrication model of the 3D networks with orthogonal hybrid AgNRs/NPs arrays. (**c**) Transmission electron microscopy (TEM) image of the hybrid AgNRs/NPs showing continuous lattice fringes. Scale bar in the inset is 2 nm. (**d**)The variation in contact angle of water on AJP-derived films with different print layers, showing changing deposition behaviors. (**e**) The ordered migration and assembly of hybrid AgNRs/NPs on pre-deposited Ag traces with increasing surface energy.

Figure 1a illustrates a schematic of the AJP technique for printing 3D networks of hybrid AgNRs/NPs as well as the sensor fabrication procedures. During the AJP procedure, aerosol droplets carrying AgNRs/NPs were transported to the nozzle by a carrier gas and then confined by a sheath gas. The aerosol mist flow was concentrated and deposited on a clean glass substrate. The mist of aerosol droplets confined within the sheath gas can be thought of as a multiple quasi-parallel droplet flow, and the multiple mono-directional surface tension derived from small meniscus-shaped liquid/solid/gas three-phase contact line (TCL) dominates the alignment of Ag species [39,40]. The Ag species were observed to have moved to the edges of the printing traces due to the coffee-ring effect, accompanied by the complete evaporation of the solvent (Figure 1b). This migration exemplifies the oriented assembly of Ag species near the edges, which is helpful for the formation of ordered networks with uniform gaps (Figure 1c,d). The printing quality is determined by the focus ratio (FR), which is defined as the ratio of the sheath gas flow rate to the carrier flow rate [41]. The width of the printing traces increased according to the carrier flow while the sheath gas flow was held constant. When the FR is 2, the width of the printing traces is around 100 μm and the diameter of a single track is about 10 μm (Figure S2). In accordance with this parameter condition, the structurally integrated 3D networks were progressively printed on a glass substrate using various orthogonal printing passes with pre-designed graphic data. Following that, a PVA solution was cast onto the printed patterns, and the polymerized film was peeled away from the glass substrate (Figure 1e). The liquid polymer solution infiltrated the network’s interconnected pores when it was cast onto the network. Polymerization of the penetrating polymer would improve the mutual overlap of Ag species, hence enhancing the electrical conductivity of the 3D conductive networks. Figure 2 depicts the fabricated flexible strain sensor with structurally integrated 3D conductive networks. Due to the PVA elastomer-encased 3D networks, the sensors are durable and exhibit no fractures after repeated 25% stretching.

Alternative orthogonal AJP printing passes enabled the creation of 3D networks, and as a result, the microstructure was reliant on the printing layers. The generated 3D networks-embedded films were rather dense after polymer penetration. The film’s thickness increased proportionally to the number of printing layers, while the length of the AgNRs remained constant (Figure S1f). The thickness of the 15-layered film is calculated to be 562.1 nm (Figure S3). The AgNRs/NPs encased in the PVA elastomer became more visible in the SEM image as the printing layers increased, which can be attributed to poor permeation of liquid polymer solution into the interconnected pores (Figure 2a). It is obvious that crossed AgNRs/NPs could be visible on the top surface of flexible sensors in the case of thick films (Figure 2b). The AgNRs/NPs were stacked orthogonally, resulting in the formation of highly conductive pathways. The AgNPs were discovered to be solidly anchored to the surface of the Ag nanorod with a virtually continuous lattice fringe, indicating the formation of a hybrid structure (Figure 2c) [42]. The mechanical stability of the flexible sensors may benefit from the 3D networks of hybrid AgNRs/NPs enclosed in the PVA elastomer. The production of hybrid AgNRs/NPs may have resulted from the microreactions that took place inside the microscale droplets [30]. The solvent in the deposited droplets is susceptible to evaporation on heat substrate, and the inward capillary force would compress Ag pieces from multiple directions, with AgNPs attaching to the nanorods with high surface energy in order to reduce the total energy [43]. In addition, the accumulation of Ag species towards the edges of the printed traces would exacerbate the generation of hybrid structures.

Despite the formation of 3D networks, the distance between neighboring hybrid AgNRs/NPs was considerably reduced, which can be attributed to variations in wetting throughout the multiple printing passes. After initial printing of hybrid AgNRs/NPs, the deposited surface for aerosol droplets became more hydrophilic as surface tension increased. On the pre-deposited surface, the contact angle reduced dramatically (Figure 2e). The aerosol droplets deposited on the clean substrate would be pinned, and the coffee-ring effect would occur in the evaporating droplets, causing the Marangoni reflux to be overcome by a strong capillary flow [44]. It is capable of dragging the suspended Ag species to deposit completely at the droplet’s periphery, so enabling the rail-like patterns. The pre-deposited PVA-capped Ag patterns were hydrophilic to the succeeding drops, allowing for the measurement of a small contact angle. In this situation, the droplets’ deposition behavior would shift from pinned mode to spreading mode. For a droplet that spreads, the Ag species would be carried outward by the sap flow and deposit when the solvent evaporates. As the solvent tends to entirely evaporate prior to the complete deposition of the solute, the deposition at the borders is thicker than in the middle. For a thicker film, the increased surface energy would promote the droplets spreading and make it impossible to distinguish the boundaries (Figure 2d). Consequently, the deposited films would be denser and more uniform, although the orthogonal arrangement could still be distinguished.

### 3.2. Strain Sensitivity Investigation of Hybrid Sensors

**Figure 3:**
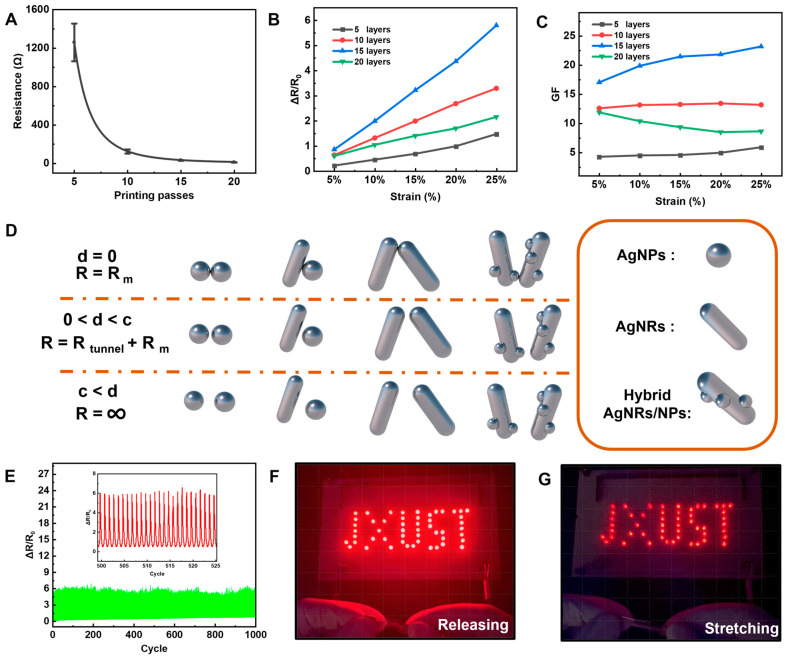
Sensing performance and mechanism investigation. (**A**) Variation in resistance of the 3D networks-based sensor as a function of printing passes. (**B**) Relative resistance changes of the sensors with different printing layers under stretching. (**C**) GF changes of the sensors with different printing layers under stretching. (**D**) The model of different electrical interconnections between AgNRs and AgNPs. (**E**) Cyclic stretching and releasing tests of the sensors. (**F**,**G**) Bright variation in brightness of the connected LEDs under releasing and stretching states.

To evaluate the quality of the 3D conductive networks of the sensors, the dependence of printing layers on resistance has been explored. As seen in Figure 3a, the resistance initially reduced dramatically and then remained somewhat steady as the number of printing layers increased. As for the 5-layered sensor, the resistance reaches a maximum of 1259.3 Ω. The conductive networks with reduced resistance fluctuation were well developed with the production of a dense film, and a low value of resistance (11.3 Ω) could be obtained for the 20-layered device. From the sensors with various layer configurations, it has been estimated that the relative resistance varies during stretching from 0% to 25% (Figure 3b). When all of the sensors are stretched, the relative resistance changes linearly. The drastically increasing slope as the number of layers increases implies improved sensing performance [45]. The gauge factor (*GF*) is an important indicator for sensors that can be calculated by:

$$GF=\frac{△R}{\varepsilon R}=\frac{R-R_{0}}{R_{0}}/\frac{L-L_{0}}{L_{0}}$$

where *R*_0_ is the initial resistance of the sensor, and *R* is a resistance measured during stretching, ε is the rate of change in length, *L*_0_ is the initial length of the sensor, and *L* is the length during stretching. Figure 3c illustrates the changes in *GF* at various strain ranges for sensors with different printing layers. For all sensors with variable-thickness layers, the *GF*s vary linearly with increasing strain ranges. The linearity of the strain sensor is crucial since it enables the strain rate to be retrieved more readily after the resistance change is known [46]. The linearly altered *GF* may have come from the well-established 3D conductive networks, and there was no discernible positional shift of hybrid AgNRs/NPs within the investigated strain ranges. It is well known that resistance change is strongly associated with positional shift of conductive components [47]. During stretching, an external force extends the sensor, resulting in a change in resistance due to the alteration of lap state between the hybrid AgNRs/NPs in the elastomers. The resistance of the sensor is composed of internal resistance and tunneling resistance of the components [18]. The tunneling resistance can be approximately described by [48]:

$$R_{tunnel}=\frac{V}{AJ}=\frac{h^{2}d}{Ae^{2}\sqrt{2m\lambda }}\exp\left(\frac{4\pi d}{h}\sqrt{2m\lambda }\right)$$

where *V* is the electrical potential difference, *J* is tunneling current density, *e* is the single-electron charge, *m* is the mass of the electron, *h* is Planck’s constant, *λ* is the height of the energy barrier, and *A* is the cross-sectional area of the tunnel. It may be concluded that the magnitude change of the tunneling resistance is only dependent on the component distance (d). When the cut-off distance (c) is reached, the resistance reaches infinity and no current flows between the components. To describe the resistance composition of strain sensors while stretching or releasing, the resistor models for each configuration are presented (Figure 3d). Three states can be identified between the hybrid AgNRs/NPs in the working sensor. The resistance is the material’s inherent impedance when there is no gap between the materials. When the distance between the hybrid AgNRs/NPs is higher than zero and less than the cut-off distance, the resistance value is the material’s resistance and the sum of the tunnel resistance. When the distance exceeds the cut-off value, the resistance value becomes infinite. Excessive strain range would result in the destruction of conductive channels due to the fragmentation of hybrid AgNRs/NPs, hence preventing significant resistance changes upon stretching. In this study, the formation of hybrid AgNRs/NPs provides sufficient and robust conductive pathways for enhancing the sensors’ sensitivity. Under the tested strain ranges, the linear variation in *GF* indicates that the sensors’ cut-off distance was not reached. As the number of layers increases from 5 to 15, the *GF* exhibits a clear rising trend due to the incremental optimization of the 3D conductive network after numerous printing passes. Nonetheless, the 20-layered sensor had a lower *GF* than its 15-layered counterpart. The decrease in *GF* can be attributed to the fact that the PVA elastomer-enhanced 3D networks were not properly established due to insufficient polymer penetration in the thick and dense films. The 15-layered sensor had the best resistance change rate and *GF* over a 25% strain range.

By constructing a closed circuit consisting of the strain sensor, a source of direct current, resistances, and LEDs, the strain sensor’s sensitivity with PVA elastomer-enhanced 3D networks could be demonstrated intuitively (Figure S4). When the sensor is stretched, it functions as a rheostat, and the resistance value changes. While the voltage across the circuit remains constant, stretching or releasing the sensor will result in a decrease in current, which will alter the brightness of the LEDs, as shown in Figure 3f,g (Movie S1). The change in resistance of this sensor displays good repeatability during cyclic stretching and releasing testing up to 1000 times (Figure 3e), indicating high mechanical reliability. Furthermore, under the circumstance of bending tests, this sensor exhibits good repeatability (Figure S5). In recent years, AJP has gained significant interest in the development of flexible strain sensors [49]. Comparatively, the sensors of the elastomer-intensified 3D networks of hybrid AgNRs/NPs have demonstrated the optimal sensing performance, as evidenced by the published works (Table S1). In this sensor configuration, the 3D network serves as a conductivity network, while the attached nanoparticles help to improve the sensor’s sensitivity. The 3D conductive networks of hybrid AgNRs/NPs were further intensified when they were encased in the elastomer.

### 3.3. Wearable Healthcare Hybrid Sensors

**Figure 4:**
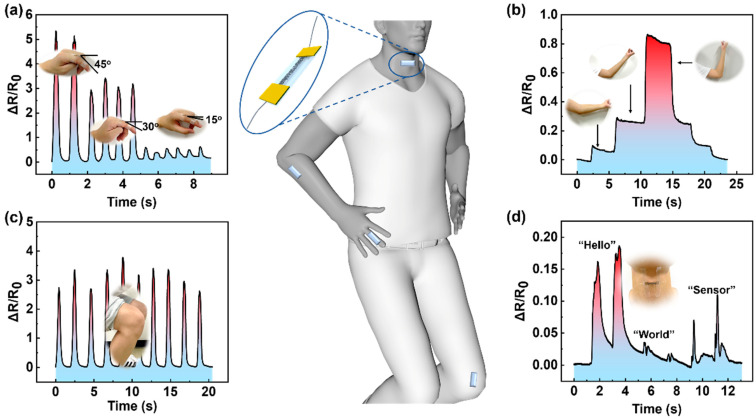
Wearable healthcare monitoring devices. (**a**) The relationship between change rate of resistance and finger bending degree with a strain sensor bonded to the finger joint. (**b**,**c**) The change rate of resistance varies with the joints movements of elbow and knee. (**d**) Demonstration of personal voice identification application.

Currently, there is a growing demand for simple and trustworthy healthcare monitoring solutions for health evaluations and disease diagnosis [50,51]. The developed strain sensors have been utilized as wearable healthcare electronic devices to detect actual human joint movements. Since the elastomer-based sensor can be easily bent and stretched, the strain caused by joint movement is predominantly along the rear side, which is conveyed to the sensor and causes its resistance to change. As a proof-of-concept, the elastomer-based sensor is bonded securely and conformably to the finger joint, elbow, knee, and throat surface. Each joint movement can elicit a constant and reliable response from the developed strain sensors. When evaluating finger movements, it was discovered that the degree of finger bending had a significant impact on the change rate of resistance. A significant bending would lead to a rapid change in resistance. When the bending angles are 15^o^ and 45^o^, respectively, the change rate can range from about 1 to 5 (Figure 4a). Similar phenomena have been seen when examining elbow and knee movements. The change rate of resistance of the sensors returns to zero when the strain is released, suggesting a quick response to the delicate movements of the joints (Figure 4b,c). When the sensor was tested, it was discovered to be sensitive to pronouncing phrases such as “hello,” “world,” and “sensor,” which created varied small vibrations (Figure 4d). As a result, varied resistance change rates could be detected, and the constructed flexible strain sensors with great sensitivity will have enormous promise for applications in wearable and healthcare electronics.

## 4. Conclusions

In conclusion, we demonstrated a structurally integrated 3D conductive network consisting of orthogonally assembled hybrid AgNRs/NPs embedded into a polymer matrix by regulating the deposition behaviors of aerosol droplets during an efficient aerosol jet printing process. The hybrid AgNRs/NPs enhance interfacial conduction and mechanical properties during stretching. The change in wetting from pinned mode to spreading mode of deposited quasi-parallel aerosol droplets supports the formation of a graded 3D network of hybrid AgNRs/NPs, which is advantageous for conformal contact with the skin for a rapid sensitive response. In response to stretching that was not limited to 25%, the relative resistance showed a linear variation. As a conformal/skin-attachable device application, our flexible strain sensor was used to detect actual human joint movements, allowing for intimate contact with human skin and resulting in reliable and rapid changes with finger, elbow, and knee movements. Our flexible strain sensor device was used for a personal voice identification application as a proof-of-concept demonstration. The sensor was able to recognize user-pronouncing phrases and respond appropriately. These stretchable and flexible devices comprised of hybrid nanostructures can be further investigated for a variety of potential applications, including wearable sensors and conformal healthcare devices.

## Data Availability

Data sharing is not applicable to this article.

## Supplementary Materials

The following supporting information can be downloaded at: https://www.mdpi.com/article/10.3390/nano13010181/s1, Figure S1. Ink formulation for aerosol jet printing of hybrid Ag-based sensors; Figure S2. The dependence of focus ratio (FR) on the printing patterns of silver species (5 passes); Figure S3. The relationship between film thickness and printing passes, indicating linearly increasing upon layers. Figure S4. A circuit diagram consisting of the strain sensor, resistances and LEDs for testing the sensor’s sensitivity showing variable brightness of the LEDs upon stretching or releasing. Figure S5. Cyclic bending tests of the sensors. Table S1. Summary and comparison of AJP-derived strain sensors reported.; Movie S1: Variable brightness of the LEDs upon stretching or releasing of the structurally integrated 3D conductive networks-based flexible strain sensors. References [52,53,54,55,56] are cited in supplementary materials.
