# Supplementary material for: Droplets Patterning of Structurally Integrated 3D Conductive Networks-Based Flexible Strain Sensors for Healthcare Monitoring
Source: Nanomaterials (Basel). 2022 Dec 30;13(1):181. doi: 10.3390/nano13010181 (PMC9824308; doi:10.3390/nano13010181)
Supplement: Supplementary file 1 [file nanomaterials-13-00181-s001.zip › Supporting Information.pdf]

nanomaterials

Supplementary Materials

Droplets Patterning of Structurally Integrated 3D Conductive Networks-Based Flexible Strain Sensors for Healthcare Monitoring

Yang Zhang [1], Danjiao Zhao [1], Lei Cao [1], Lanlan Fan [1], Aiping Lin [1], Shufen Wang [1], Feng Gu [1,2,*] and Aibing Yu [1,3,*]

1 Laboratory of Advanced Materials & Manufacturing (LAMM), Nanchang Key Laboratory for Advanced Manufacturing of Electronic Information Materials and Devices, International Institute for Innovation, Jiangxi University of Science and Technology, Nanchang 330013, China
2 Institute for Process Modelling and Optimization, Jiangsu Industrial Technology Research Institute, Suzhou 215123, China
3 Department of Chemical Engineering, Monash University, Melbourne, VIC 3800, Australia
* Correspondence: feng.gu@jxust.edu.cn (F.G); aibing.yu@monash.edu (A.Y)

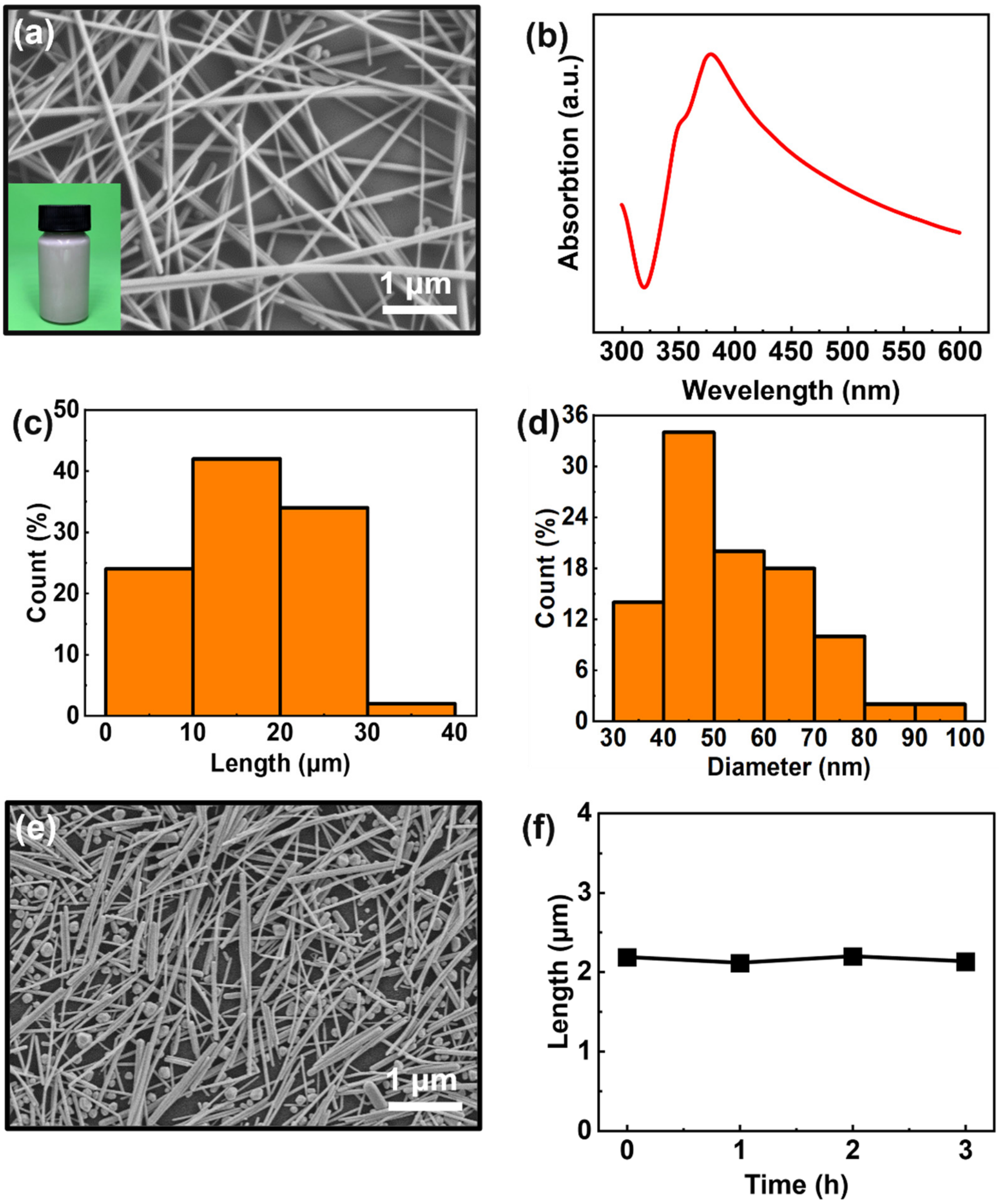

Figure S1. Ink formulation for aerosol jet printing of hybrid Ag-based sensors. (**a**) SEM image of precursor Ag nanowires. (**b**) UV absorption spectrum of Ag nanowire aqueous solution. (**c**) Length distribution of the precursor Ag nanowires. (**d**) Diameter distribution of the precursor Ag nanowires. (**e**) SEM image of Ag nanoparticles and silver nanorods derived from Ag nanowires fragment. (**f**) Relationship between ultrasonic nebulization duration and the average length of silver nanorods.

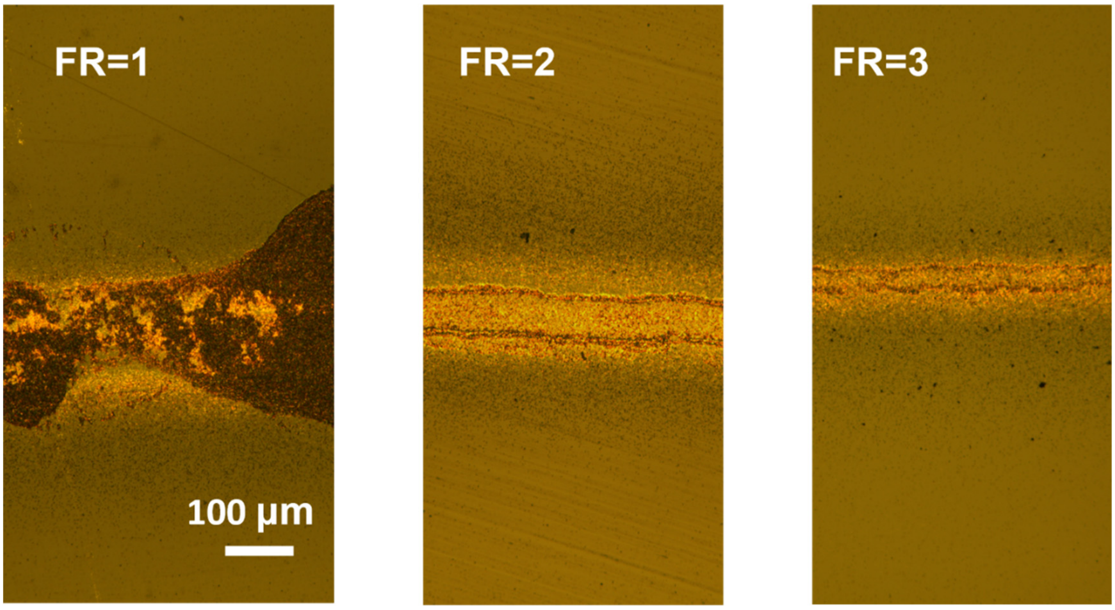

Figure S2. The dependence of focus ratio (FR) on the printing patterns of silver species (5 passes).

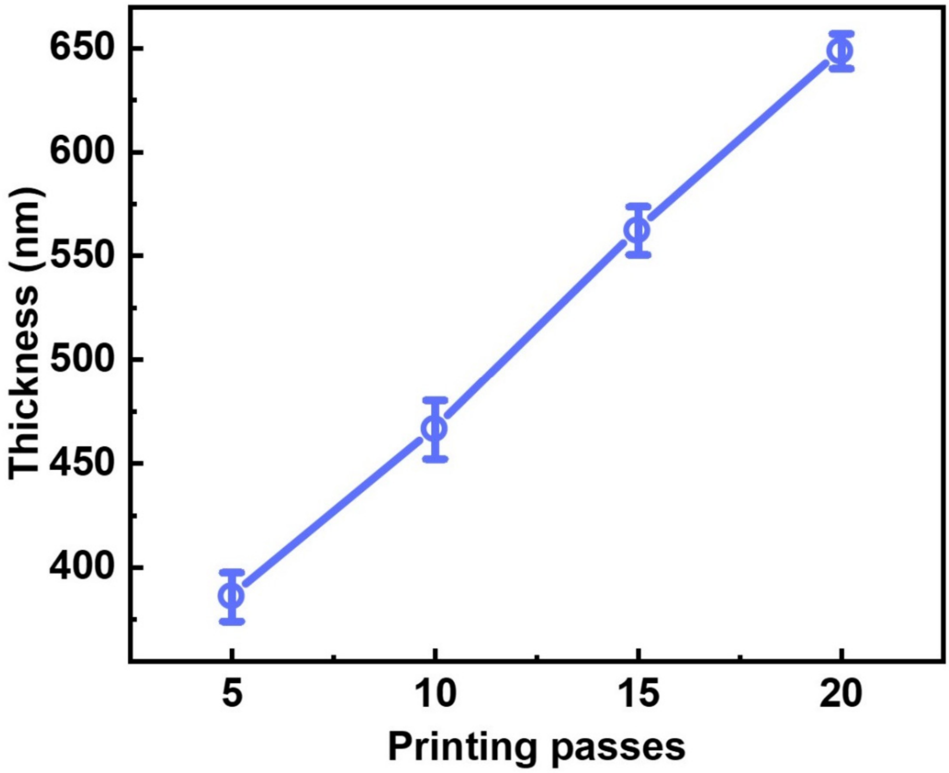

Figure S3. The relationship between film thickness and printing passes, indicating linearly increasing upon layers.

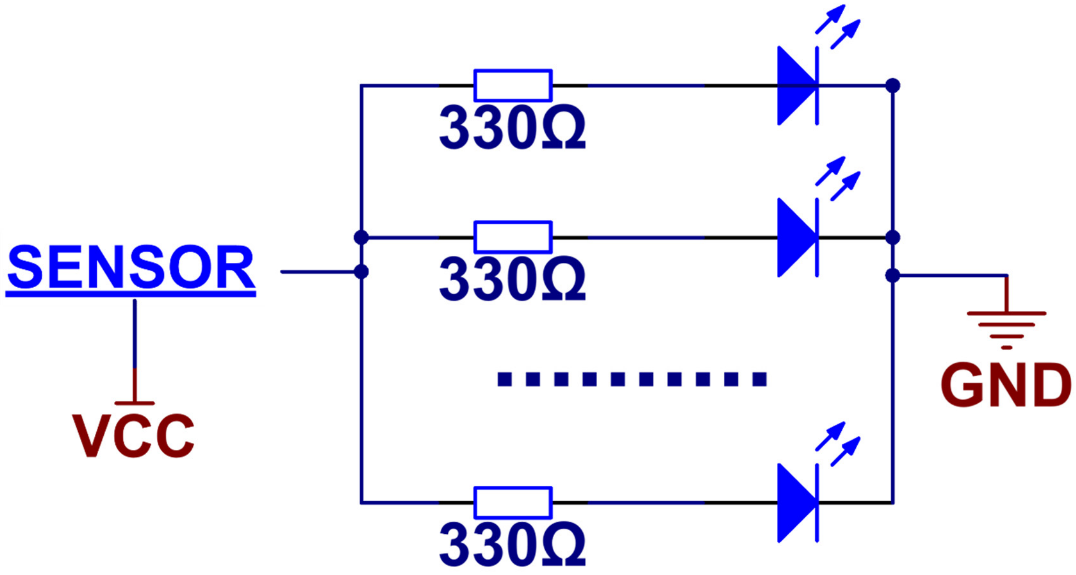

Figure S4. A circuit diagram consisting of the strain sensor, resistances and LEDs for testing the sensor's sensitivity showing variable brightness of the LEDs upon stretching or releasing.

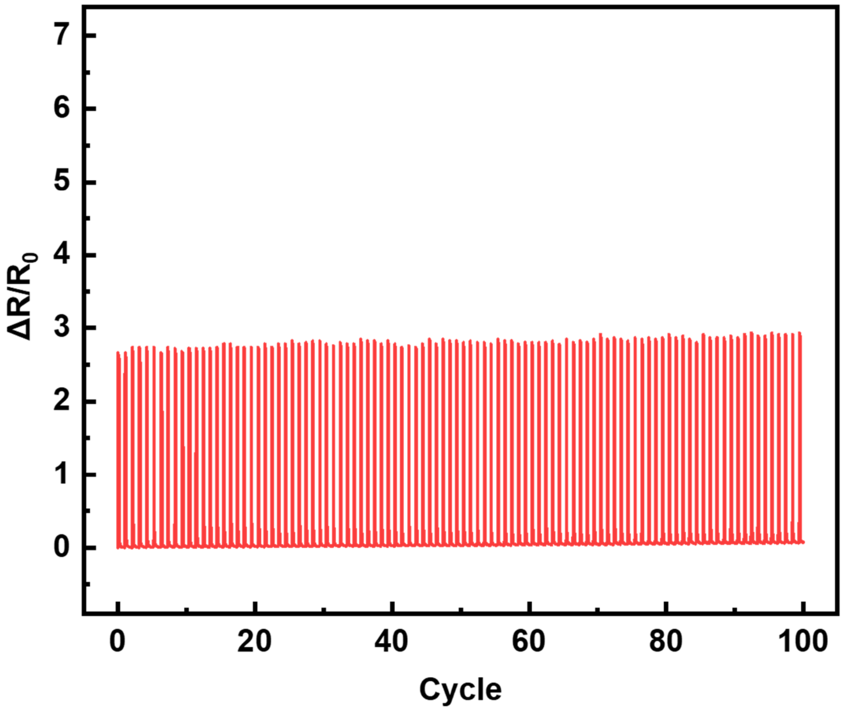

Figure S5. Cyclic bending tests of the sensors.

Table S1. Summary and comparison of AJP-derived strain sensors reported.

Material	Strain range	Gauge factor	Postprocessing	Ref.
**AgNRs AgNPs / PVA**	25%	23.18		**This work**
**AgNWs / Pi**	4%	7.5	Anneal	[1]
**AgNPs/ Kapton**	1.5%	9-10	Anneal	[2]
**MXene-Graphene / Kapton**		3.7	Anneal	[3]
**AgNPs /PVC**	0.25%	2.57	Laser sintering	[4]
**CNT-AgNPs /Glass fiber**		1.71	Anneal	[5]

References

1. Herbert, R.; Lim, H.-R.; Yeo, W.-H. Printed, Soft, Nanostructured Strain Sensors for Monitoring of Structural Health and Human Physiology. *ACS Appl. Mater. Interfaces* **2020**, *12*, 25020–25030, doi:10.1021/acsami.0c04857.
2. Agarwala, S.; Goh, G.L.; Yeong, W.Y. Aerosol Jet Printed Strain Sensor: Simulation Studies Analyzing the Effect of Dimension and Design on Performance (September 2018). *IEEE Access* **2018**, *6*, 63080–63086, doi:10.1109/ACCESS.2018.2876647.
3. Saeidi-Javash, M.; Du, Y.; Zeng, M.; Wyatt, B.C.; Zhang, B.; Kempf, N.; Anasori, B.; Zhang, Y. All-Printed MXene–Graphene Nanosheet-Based Bimodal Sensors for Simultaneous Strain and Temperature Sensing. *ACS Appl. Electron. Mater.* **2021**, *3*, 2341–2348, doi:10.1021/acsaelm.1c00218.
4. Borghetti, M.; Serpelloni, M.; Sardini, E. Printed Strain Gauge on 3D and Low-Melting Point Plastic Surface by Aerosol Jet Printing and Photonic Curing. *Sensors* **2019**, *19*, 4220, doi:10.3390/s19194220.
5. Siddique, S.; Park, J.G.; Andrei, P.; Liang, R. M3D Aerosol Jet Printed Buckypaper Multifunctional Sensors for Composite Structural Health Monitoring. *Results in Physics* **2019**, *13*, 102094, doi:10.1016/j.rinp.2019.02.030.
